# RSL3 enhances ROS-mediated cell apoptosis of myelodysplastic syndrome cells through MYB/Bcl-2 signaling pathway

**DOI:** 10.1038/s41419-024-06866-5

**Published:** 2024-07-02

**Authors:** Li Liu, Chaoying Yang, Lin Zhu, Yanyan Wang, Fuxiang Zheng, Long Liang, Pengfei Cao, Jing Liu, Xu Han, Ji Zhang

**Affiliations:** 1 Department of Clinical Laboratory, The Affiliated Nanhua Hospital, University of South China, Hengyang, 421001 Hunan China; 2 Molecular Biology Research Center & Center for Medical Genetics, School of Life Sciences, Central South University, Changsha, 410078 Hunan China; 3 Department of Dermatology, Xiangya Hospital, Central South University, Changsha, 410078 Hunan China; 4 Department of Clinical Laboratory, Shenzhen Traditional Chinese Medicine Hospital, Shenzhen, 518033 Guangdong China; 5 Department of Hematology, Xiangya Hospital, Central South University, Changsha, 410078 Hunan China; 6 MOE Key Laboratory of Rare Pediatric Diseases, Hengyang Medical School, University of South China, Hengyang, 421001 Hunan China

**Keywords:** Myelodysplastic syndrome, Diagnostic markers

## Abstract

Myelodysplastic syndromes (MDS) are clonal hematopoietic malignancies and seriously threaten people’s health. Current therapies include bone marrow transplantation and several hypomethylating agents. However, many elderly patients cannot benefit from bone marrow transplantation and many patients develop drug resistance to hypomethylating agents, making it urgent to explore novel therapy. RSL3 can effectively induce ferroptosis in various tumors and combination of RSL3 and hypomethylating agents is promising to treat many tumors. However, its effect in MDS was unknown. In this study, we found that RSL3 inhibited MDS cell proliferation through inducing ROS-dependent apoptosis. RSL3 inhibited Bcl-2 expression and increased caspase 3 and PARP cleavage. RNA-seq analysis revealed that MYB may be a potential target of RSL3. Rescue experiments showed that overexpression of MYB can rescue MDS cell proliferation inhibition caused by RSL3. Cellular thermal shift assay showed that RSL3 binds to MYB to exert its function. Furthermore, RSL3 inhibited tumor growth and decreased MYB and Bcl-2 expression in vivo. More importantly, RSL3 decreased the viability of bone marrow mononuclear cells (BMMCs) isolated from MDS patients, and RSL3 had a synergistic effect with DAC in MDS cells. Our studies have uncovered RSL3 as a promising compound and MYB/Bcl-2 signaling pathway as a potential target for MDS treatment.

## Introduction

Myelodysplastic syndromes (MDS) are highly heterogeneous clonal hematological malignancies characterized by ineffective hematopoiesis, intractable cytopenia, and high risk of transforming to acute myeloid leukemia (AML) [1, 2]. MDS incidence increases with age, especially in individuals older than 70 years [3]. Hematopoietic cell transplantation could cure MDS patients, but fewer than 10% of them received this treatment [1]. Currently, hypomethylation chemotherapy and immunomodulatory drugs are the major clinical treatment strategies [4]. However, many patients have intrinsic drug resistance or develop acquired resistance [5]. Therefore, there is an urgent need to explore the pathogenesis of MDS and develop novel therapy.

Apoptosis is an important type of programmed cell death. Previous studies have shown that apoptosis plays a critical role in the pathophysiology of MDS [6]. Clonal marrow cells isolated from patients with early MDS are prone to apoptosis, whereas cells isolated from patients with advanced MDS can evade proapoptotic signals [7].

Reactive oxygen species (ROS) are the main type of free radical present in biological systems. ROS are usually produced in mitochondria [8, 9]. Low levels of ROS result in the initiation, development, and progression of malignancies, whereas high levels of ROS can lead to apoptosis of cancer cells [10, 11]. Many drugs exert antitumor effects by activating ROS-dependent apoptosis. For example, celastrol was reported to induce apoptosis via Prdx2-mediated ROS accumulation in gastric cancer [12]. Sodium selenite and selenomethionine can individually induce ROS-mediated apoptosis in myelodysplastic cells [13].

MYB has been identified as a transcription factor crucial for hematopoiesis and erythropoiesis [14]. MYB expression is aberrantly increased and plays important roles in many hematopoietic malignancies including MDS [15, 16]. In adult zebrafish, MYB hyperactivity leads to myelodysplasia and organ infiltration and promotes progression to AML [15]. These studies indicated that MYB is a potential therapeutic target in MDS. Bcl-2 was downstream antiapoptotic protein of MYB. Notably, Bcl-2 is highly expressed in various hematological malignancies, including AML and MDS [17]. High-risk MDS have less apoptosis associated with increased expression of the pro-survival Bcl-2-related proteins [18]. Targeted inhibition of Bcl-2 proteins is a rational therapeutic option for hematological malignancies that are dependent on antiapoptotic Bcl-2 proteins [19]. Bcl-2 inhibition in combination with hypomethylating agents (HMA) has been proved to be effective in AML [20]. Venetoclax (a Bcl-2 inhibitor) combinations was superior to chemoimmunotherapy in chronic lymphocytic leukemia (CLL) [21]. Impressive responses to venetoclax have been observed in MDS patients, indicating the excellent clinical application potential of this drug [22].

RSL3 is a known ferroptosis inducer that directly inhibits GPX4, constituting a novel therapeutic approach for various cancers [16, 23, 24]. However, the antitumor effect of RSL3 in MDS is not yet clear. In this study, we found that RSL3 induces apoptosis to inhibit MDS cell proliferation, and this effect was dependent on ROS production. Importantly, MYB was significantly downregulated after RSL3 treatment. Rescue experiments further showed that RSL3 promoted MDS cell apoptosis through MYB/Bcl-2 pathway. In vivo, RSL3 suppressed the growth of MDS xenograft tumor. Notably, RSL3 inhibits the viability of mononuclear cells in MDS patients and has a synergistic effect with decitabine. Our study provides a novel therapeutic target and a potential agent for MDS treatment.

## Materials and methods

### Cell lines

MDS cell lines (MDS-L and SKM-1) were obtained from ATCC (Manassas, USA). RPMI-1640 medium (Gibco, USA) supplemented with 10% FBS (EveryGreen, China) was used to maintain MDS-L and SKM-1 cells. MDS-L cells additionally required supplementation with IL-3. Cells were identified by short tandem repeat profiling and cultured at 37 °C in an atmosphere containing 5% CO_2_.

### Cell viability assay

Cell viability was measured using a Cell Counting Kit-8 (CCK-8) assay (Zeta-Life, Menlo Park, USA). After cells were treated with RSL3, 10% CCK-8 solution was added to each well, and the cells were incubated for 2 h. The absorbance of the samples was then measured at 450 nm using a microplate reader (BioTek, Winooski, USA).

### Soft agar clonogenic assay

3 × 10^3^ cells were resuspended in 0.33% agar and then seeded into a 12-well plate. After 2 weeks, images of the 12-well plate were acquired using a Gene Genius Bio-imaging System (Bio-Rad, Hercules, USA), and the colonies were counted using ImageJ (National Institutes of Health, Bethesda, USA).

### Protein extraction and western blotting

Cell lysates were prepared using radioimmunoprecipitation assay buffer supplemented with protease and phosphatase inhibitors [25]. Protein concentrations were quantified using a Bicinchoninic Acid Protein Assay Kit (Thermo Fisher Scientific, Waltham, USA). Equal quantities of proteins were separated using sodium dodecyl sulfate-polyacrylamide gel electrophoresis and transferred onto nitrocellulose membranes (Santa Cruz Biotechnology, Dallas, USA). The membranes were blocked by 5% non-fat dry milk and incubated with primary antibodies against cleaved caspase 3 (1:1000, TA7022S, Abmart, China), MYB (1:2000, 05175, Sigma-Aldrich, USA), Bcl-2 (1:500, AF301143, AiFang biological, China), PARP (1:1000, T40050, Abmart, China), γ-H2AX (1:1000, 10856-1-AP, Cell signaling Technology, USA) and GAPDH (1:1000, sc-32233; Santa Cruz Biotechnology, USA) at 4 °C overnight. The HRP-conjugated secondary antibodies were used at 1:3000 dilution for 1.5 h at room temperature. Signals were detected by ECL HRP substrate (Sigma-Aldrich, USA).

### Quantitative real-time polymerase chain reaction (qRT–PCR)

Total RNA was extracted with TRIzol® reagent (Vazyme, Nanjing, China) according to the manufacturer’s instructions. RNA was then reverse transcribed into cDNA using the HiScript® Q RT SuperMix for qPCR Kit (Vazyme, Nanjing, China). qRT–PCR was performed using a Mastercycler® ep realplex system (Eppendorf, Hamburg, Germany), and mRNA expression levels were then calculated using the 2^–ΔΔCT^ method. The primer sequences were as follows: MYB forward: GCAGGTGCTACCAACACAGA, reverse: CGAGGCGCTTTCTTCAGGTA and GAPDH forward: GGAGCGAGATCCCTCCAAAAT, reverse: GGCTGTTGTCATACTTCTCATGG.

### Cell apoptosis assay

Apoptosis was measured using an Annexin V-FITC/PI Apoptosis Kit (Vazyme, Nanjing, China) according to the manufacturer’s instructions. In brief, cells were harvested and washed with DPBS buffer and resuspended in 100 μL binding buffer. Annexin V-FITC (5 μL) and propidium iodide (PI; 5 μL) were then added, and the cell suspension was incubated in the dark for 10 min. The fluorescence intensity was measured by flow cytometry (FACSCalibur, BD Biosciences, USA).

### Cellular thermal shift assay

MDS-L cells were treated by DMSO or RSL3 (Selleck, USA) for 1 h. The cells were washed for three times with precooled PBS and resuspended with 50 μL PBS. The cells were divided into three groups and separately put into water bath at 37 °C, 42 °C, 47 °C. 3 min later, the cells were frozen with liquid nitrogen. The frozen cells were thawed on the ice. Repeated freezing and thawing for three times to completely lyse the cells. Then cell lysis was harvested and used for western blot analysis.

### Myelodysplastic syndrome xenograft mouse model

Hairless female NCG mice, 4–6 weeks, were purchased from GemPharmatech (Nanjing, China). And investigators were blinded to the randomization. Mice were subcutaneously inoculated in the flanks with 8 × 10^6^ SKM-1 cells in 100 μL of DPBS. When the tumors were measurable, the mice were randomized into two groups (sample size: five mice per group) and were treated with vehicle (10% DMSO, 40% PEG300, 5% Tween-80, and 45% normal saline, control group) or RSL3 (20 mg/kg) by intraperitoneal injection. Tumor volume and body weight were checked every 2 days. Mice were euthanized at the end of the experiment. Tumors were quickly exfoliated, and their weight was recorded along with the group category, and photographed after the arrangement. Tumors were fixed with 10% formaldehyde and embedded in paraffin. Hematoxylin and eosin (H&E) staining, immunofluorescence staining, and a TUNEL assay were performed according to standard protocols. Images were acquired using a Nikon fluorescence microscope (Nikon Corporation, Japan).

### Isolation of primary human bone marrow cells from patients

All samples from human MDS patients and animal models used in the study were ethically approved by the Institutional Review Board Committee on Human Experimentation and Animal Care of Xiangya Hospital of Central South University and processed according to the procedure approved by the committee. Cell viability was measured with a CCK-8 assay (Vazyme, Nanjing, China) after drug treatment following the manufacturer’s protocol.

### Statistical analysis

Prism 8 (GraphPad Software, San Diego, USA) was used to present the data in bar graphs, with all values presented as the means ± standard deviations. Comparisons between two groups were performed using an unpaired, two-tailed *t* test. *p < 0.05, **p < 0.01, ***p < 0.001, NS, no significant.

## Results

### RSL3 induces apoptosis in MDS cells

**Fig. 1:**
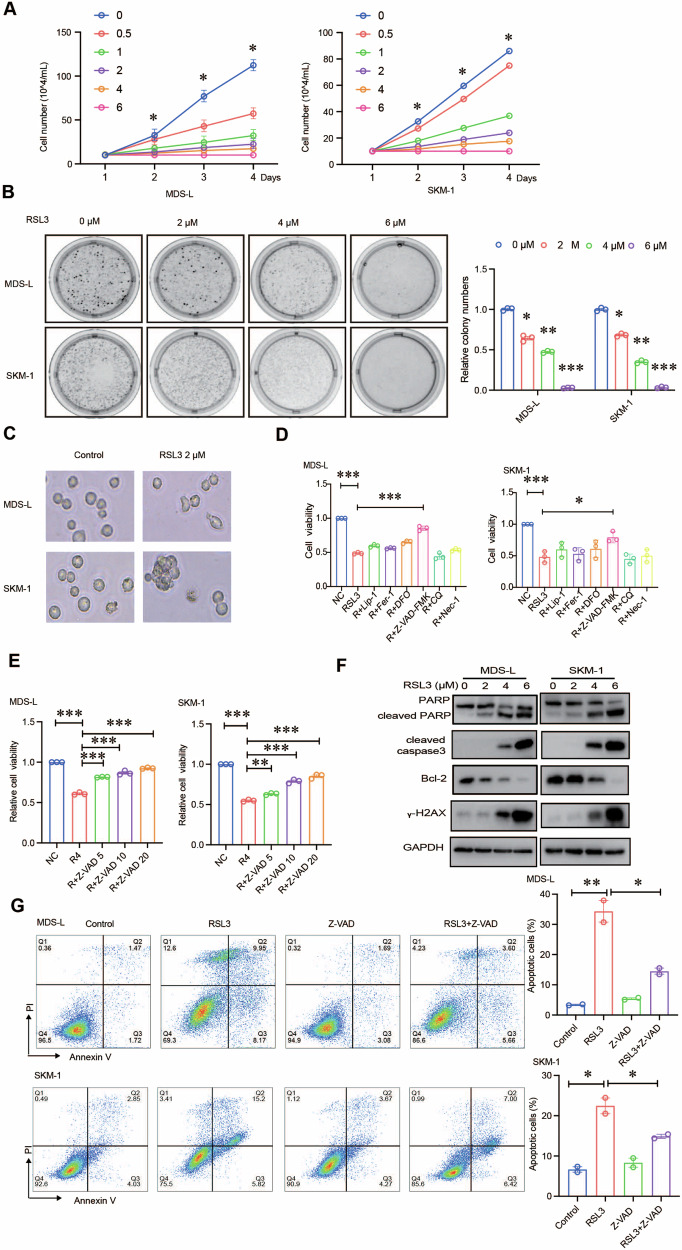
RSL3 induces apoptosis in MDS cells. **A**, **B** MDS cell proliferation was evaluated by manual counting and soft agar clonogenic assay. RSL3 significantly suppressed MDS-L and SKM-1 cell proliferation in a concentration-dependent manner. **C** Morphological changes associated with cell death after RSL3 treatment were visualized by optical microscopy. Scale bar, 100 μm. **D**, **E** MDS cells were treated with RSL3 (4 μM) in combination with Lip-1 (2 μM), Fer-1 (2 μM), DFO (100 μM), Z-VAD-FMK (10 μM), CQ (10 μM), Nec-1 (10 μM), respectively. The CCK-8 rescue assay results showed that Z-VAD-FMK notably reversed the inhibition of proliferation induced by RSL3. **F** Western blotting of the levels of apoptotic proteins, including cleaved PARP, cleaved caspase3, γ-H2AX, and Bcl-2. **G** Flow cytometric analysis showed that RSL3 increased the percentage of apoptotic cells and that Z-VAD-FMK reversed this effect after 24 h. ***p < 0.001, **p < 0.01, *p < 0.05.

To determine whether RSL3 has some roles in MDS cells, MDS-L and SKM-1 cells were treated with different concentrations of RSL3 at specific times, and cell viability was analyzed by manual counting and soft agar clonogenic assays [26, 27]. The results showed that RSL3 significantly inhibited MDS cell expansion (Fig. 1A). And soft agar clonogenic assay also demonstrated that RSL3 inhibited colony formation of MDS-L and SKM-1 cells compared with control groups (Fig. 1B). Furthermore, we explored the mechanism by which RSL3 suppresses MDS cell proliferation. It was found that RSL3 treatment led to a series of morphological changes, including reduced volume, ruptured cytomembrane, and decreased cell density, in both MDS-L and SKM-1 cells (Fig. 1C). Therefore, we speculated that RSL3 induces MDS cell death to inhibit cell proliferation. Ferroptosis, apoptosis, autophagy, and necroptosis are common types of programmed cell death [28]. To identify which kind of cell death was involved, MDS cells were treated with RSL3 alone or in combination with liproxstatin-1 (Lip-1; a ferroptosis inhibitor targeting GPX4), ferrostatin-1 (Fer-1; a ferroptosis inhibitor targeting SCL7A11), deferoxamine (DFO; a chelator), Z-VAD-FMK (an apoptosis inhibitor targeting caspase), chloroquine (CQ; an autophagy inhibitor), or necrostatin-1 (Nec-1; a necroptosis inhibitor) [29–31]. The results showed that only Z-VAD-FMK notably reversed the inhibition of proliferation induced by RSL3 in a concentration-dependent manner (Fig. 1D, E). Western blot results showed that the levels of cleaved PARP, cleaved caspase3 and γ-H2AX were increased and Bcl-2 was decreased with increasing concentrations of RSL3 in both MDS-L and SKM-1 cells (Fig. 1F), further confirming RSL3 induces apoptosis in MDS cells. Flow cytometric analysis also showed that RSL3 significantly induced MDS cell apoptosis and that Z-VAD-FMK restored cell viability (Fig. 1G). Taken together, these data showed that RSL3 suppressed the malignant proliferation of MDS cells through inducing apoptosis.

### RSL3-mediated apoptosis is dependent on ROS

**Fig. 2:**
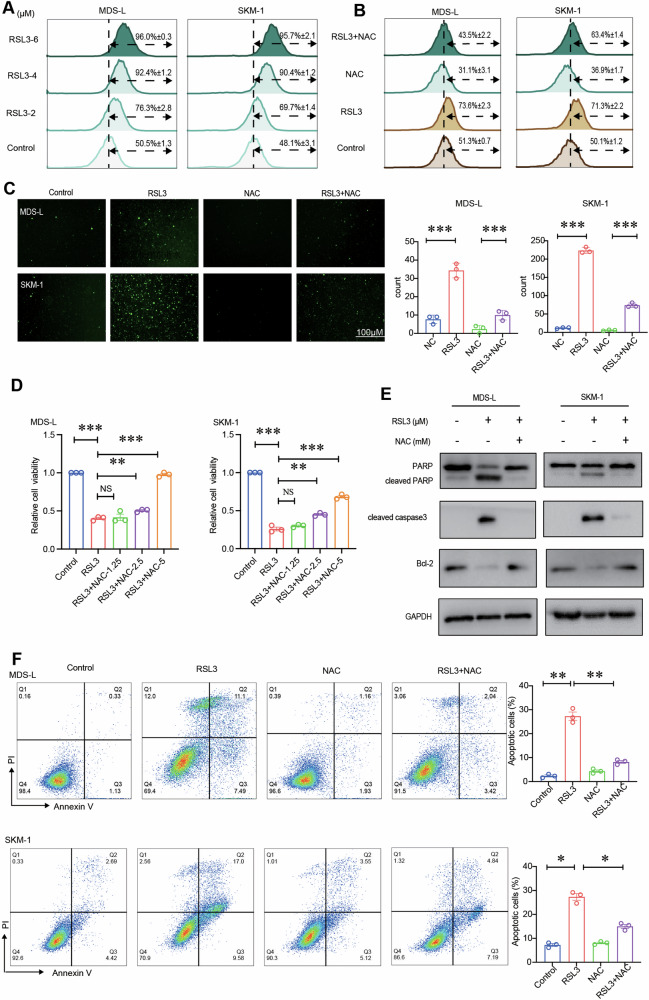
RSL3-mediated apoptosis is dependent on ROS. **A**, **B** Flow cytometric analysis after treatment with RSL3 alone or in combination with NAC. RSL3 increased ROS levels in MDS cells, and NAC (5 mM) partially reversed RSL3-triggered ROS accumulation. **C** Fluorescence microscopy analysis showed that RSL3 treatment intensified ROS fluorescence in MDS cells. NAC treatment reduced ROS fluorescence. **D** The effect of NAC on reversing the RSL3-mediated decrease in MDS cell viability was evaluated by a CCK-8 assay. **E** Cleaved PARP, Bcl-2, and cleaved caspase 3 levels were measured by western blotting after treatment with RSL3 alone or in combination with NAC. **F** Flow cytometric analysis showed that NAC reduced the RSL3-mediated apoptosis rates in MDS-L and SKM-1 cells. ***p < 0.001, **p < 0.01, *p < 0.05. NS not significant.

ROS accumulation occurs during apoptosis [32]. Based on the above results, we measured ROS levels in RSL3-treated MDS cells. MDS cells were treated for with RSL3 alone or in combination with NAC (a ROS inhibitor) and subsequently analyzed by flow cytometry. RSL3 increased ROS levels in MDS cells in a dose-dependent manner (Fig. 2A). And NAC partially reversed the RSL3-induced ROS accumulation (Fig. 2B). Furthermore, fluorescence staining also revealed that RSL3 treatment increased ROS fluorescence in MDS cells and NAC could attenuate this effect [33] (Fig. 2C). These results showed the accumulation of ROS in RSL3-treated MDS cells. At the meanwhile, cell proliferation assay showed NAC can rescue the proliferation inhibition induced by RSL3 treatment in MDS cells in a dose-dependent manner, indicating that ROS production plays an important role in the RSL3-induced MDS cell proliferation inhibition (Fig. 2D). Importantly, the levels of cleaved PARP, Bcl-2 and cleaved caspase 3, were greatly reduced after NAC treatment (Fig. 2E), and NAC reduced the RSL3-mediated apoptosis rates in MDS-L and SKM-1 cells (Fig. 2F), indicating that inhibition of ROS prevented RSL3-induced apoptosis. Thus, RSL3-triggered cell apoptosis is dependent on ROS.

### MYB is an efficacious target of RSL3-mediated apoptosis

**Fig. 3:**
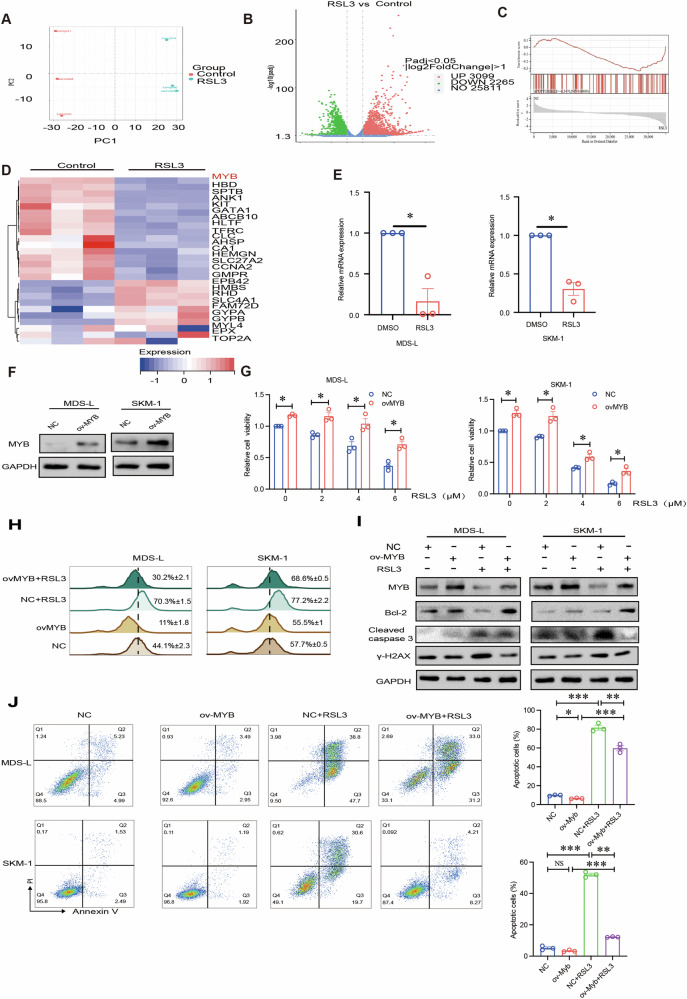
MYB is an efficacious target of RSL3-mediated apoptosis. **A** PCA plot analysis between RSL3-treated group and the control. **B** Volcano plot indicated the differential genes between the RSL3-treated group and control group. The up‐regulated genes (red), and the down‐regulated genes (green) with P adjust < 0.05 and | log2 (FoldChange)| ≥ 1. **C** GSEA enrichment analysis showed that the differentially expressed genes were significantly enriched in apoptosis pathway. **D** Venn analysis showed that MYB was involved in RSL3-mediated apoptosis in MDS after RSL3 treatment and the results were shown in the heatmap. **E** qRT-PCR showed that RSL3 downregulated expression of MYB. **F** The transfection efficiency of the MYB overexpression plasmid was determined in MDS cells. **G** The CCK-8 rescue assay showed that cell viability, which was decreased by RSL3 treatment, could be enhanced by overexpression of MYB in MDS cells. **H** Flow cytometric analysis showed that MYB overexpression rescued ROS production caused by RSL3. **I** Western blotting showed that MYB overexpression increased the Bcl-2 protein level and decreased the level of cleaved caspase 3 and γ-H2AX and reversed the effect of apoptotic protein expression caused by RSL3. **J** Flow cytometric analysis showed that MYB overexpression blocked RSL3-mediated MDS cell apoptosis after 48 h. ***p < 0.001, **p < 0.01, *p < 0.05, NS not significant.

To explore the detailed molecular mechanism of RSL3-induced cell death, RNA-seq was performed in MDS-L cells treated with RSL3 or DMSO. Principal component analysis showed clustering of triplicated samples in each group (Fig. 3A). Volcano plot showed the differential expression of all genes. Compared with control group, there were 3099 genes significantly up-regulated and 2265 genes significantly down-regulated in RSL3 treatment group (Fig. 3B). Importantly, GSEA analysis showed that apoptosis pathway was significantly enriched in RSL3-treated cells, which was consistent with RSL3’s roles (Fig. 3C). We further performed Venn diagram analysis to compare downregulated genes identified by RNA-seq with upregulated genes in MDS patients identified in a GEO dataset (GSE114869). The Venn diagram contained 26 overlapping molecules, which were visualized in a heatmap (Fig. 3D). These molecules could promote the initiation and development of MDS and be potential targets of RSL3. Our previous study found that MYB is overexpressed in clinical MDS samples compared with normal samples and that MYB regulates MDS-L and SKM-1 cell viability via PI3K-AKT pathway [4, 34]. Interestingly, RNA-seq results showed that RSL3 treatment decreased MYB expression (Fig. 3D), which was further confirmed by quantitative PCR (Fig. 3E). RNA-seq analysis showed that MYB was downregulated and ferroptosis-associated genes like EGLN1 and KEAP1 had no significant changes after RSL3 treatment (Supplemental Fig. 1A).

To further explore whether RSL3-mediated MDS cell apoptosis was related with decreased MYB levels. We did rescue assays by transfected MDS-L and SKM-1 cells with MYB overexpression plasmids. Western blot result confirmed the overexpression efficiency (Fig. 3F). And cell proliferation assay showed that MYB overexpression partially rescued the decrease in MDS cell viability caused by RSL3 (Fig. 3G). Flow cytometric analysis showed that RSL3 increased ROS production in MDS-L and SKM-1 cells and overexpression of MYB reverted this effect (Fig. 3H). More importantly, western blot results showed that MYB overexpression elevated the Bcl-2 protein level, decreased the cleaved caspase 3 and γ-H2AX, and reversed the effects of apoptotic protein expression caused by RSL3 (Fig. 3I). And the apoptosis rate in the RSL3-treated group was significantly decreased upon MYB overexpression (Fig. 3J). Taken together, RSL3-induced apoptosis in MDS cells at least partially via decreasing MYB expression.

### RSL3 reduces MYB expression via binding MYB

**Fig. 4:**
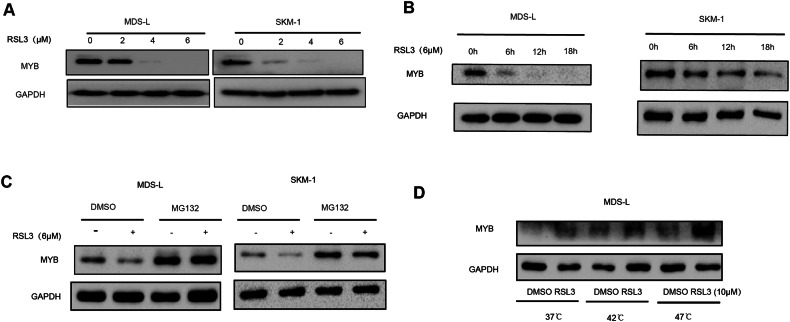
RSL3 reduces MYB expression via binding MYB. **A**, **B** Western blotting showed that RSL3 inhibited MYB expression in a concentration-dependent and time-dependent manner. **C** Western blotting indicated that RSL3-mediated MYB degradation could be rescued by MG132 after 6 h. **D** MDS cells were treated by DMSO or RSL3 at 10 μM. 1 h later, they were put into the water bath at 37 °C, 42 °C, and 47 °C. As the temperature increased, the degradation of MYB protein was reduced in the RSL3-treated group.

We further explored the mechanism of RSL3 regulating MYB expression. Western blot results showed that RSL3 treatment decreased MYB protein in dose and time-dependent manners (Fig. 4A, B). And in both MDS-L and SKM-1 cells, RSL3 treatment dramatically decreased MYB protein levels in as short as 6 h (Fig. 4B). This fast reduction of protein levels usually involves stabilization and degradation pathways. Indeed, proteasome inhibitor MG132 treatment can rescue decreased MYB protein levels led by RSL3 treatment (Fig. 4C). Furthermore, cellular thermal shift assay (CETSA) result showed that RSL3 protected MYB from thermal degradation, indicating that RSL3 can directly bind to MYB protein (Fig. 4D). In summary, these results demonstrated that RSL3 decreased MYB expression via directly binding to MYB protein and mediating its degradation via proteasome-ubiquitin pathway.

### RSL3 inhibits tumor growth in vivo

**Fig. 5:**
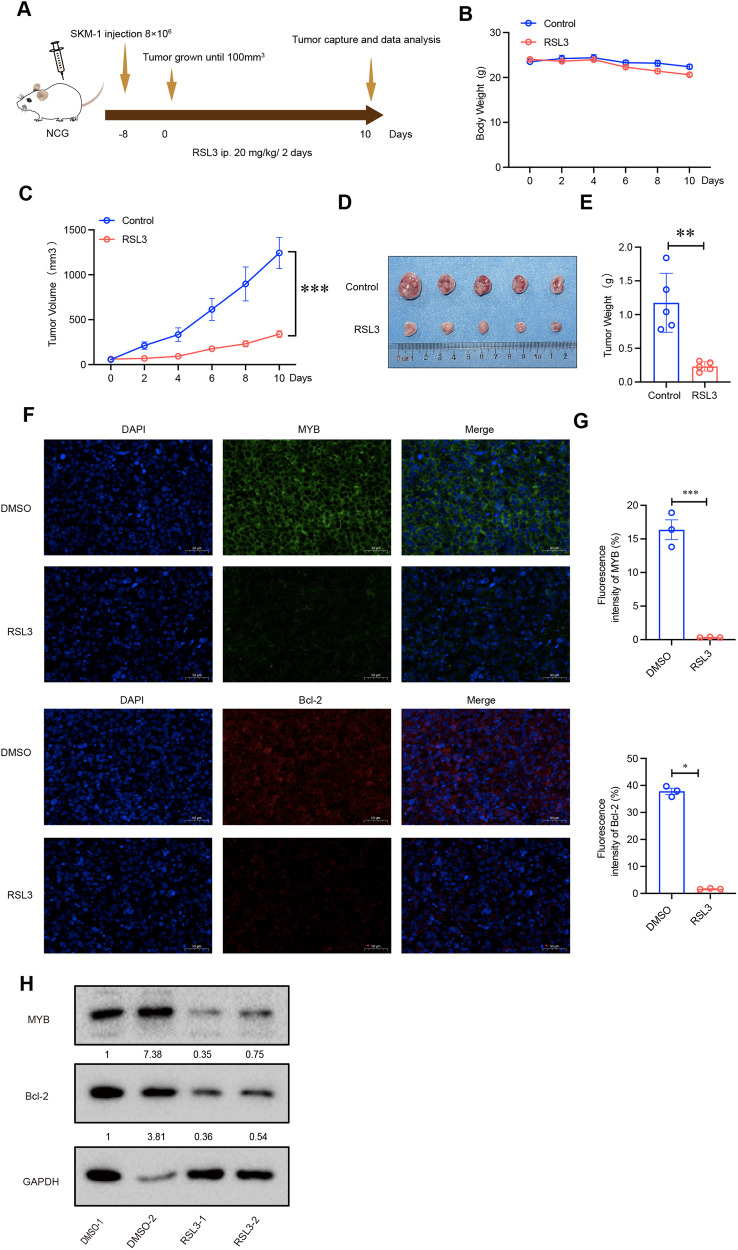
RSL3 inhibits tumor growth in vivo. **A** Schematic of the anticancer effect of RSL3 (20 mg/kg) in the SKM-1 cell subcutaneous xenograft model. **B** The body weight of mice in each group was determined every 2 days. **C** The tumor volume of mice in each group was calculated every 2 days. **D** Excised tumors on day 10. **E** Tumor weight of mice in each group. **F, G** Immunofluorescence staining showed that RSL3 treatment decreased MYB and Bcl-2 expression in the tumor tissue. Scale bar, 50 μm. **H** Western blotting showed that RSL3 decreased MYB and Bcl-2 expression in the tumor tissue. ***p < 0.001, **p < 0.01, *p < 0.05.

To further explore the antitumor role of RSL3, SKM-1 cells were inoculated subcutaneously into the flanks of NCG mice (Fig. 5A). After the tumor volume increased to 50–100 mm^3^, the mice were divided into the RSL3-treated group and the control group. RSL3 (20 mg/kg) or vehicle was administered intraperitoneally into mice once every two days [35, 36]. It was shown that tumor volume and weight were markedly smaller in RSL3-treated mice than control mice, while the body weight did not differ significantly between the RSL3-treated group and control group, indicating the safety of RSL3 administration (Fig. 5B–E). Importantly, immunofluorescence staining results showed that RSL3 treatment decreased MYB and Bcl-2 expression in the tumor tissues, which was further confirmed in western blot results (Fig. 5F–H). In conclusion, RSL3 inhibits tumor growth in vivo.

### RSL3 treatment had therapeutic efficiency in MDS patients’ derived samples

**Fig. 6:**
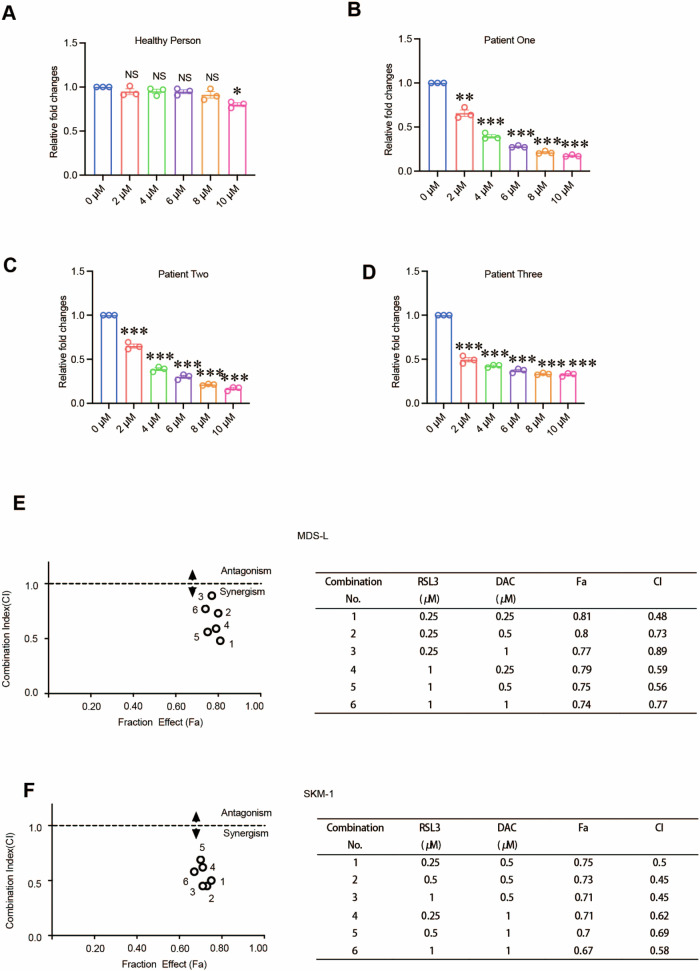
RSL3 treatment had therapeutic efficiency in MDS patients’ derived samples. **A**–**D** The CCK-8 assay results showed that RSL3 decreased cell viability in MDS patients but not healthy individuals. **E**, **F** Combination index (CI) values of RSL3 and DAC in MDS-L cells and SKM-1 cells. Both were treated with different concentrations of RSL3, DAC, or RSL3 plus DAC for 48 h. Cell viability was assessed using the CCK-8 assay. ***p < 0.001, **p < 0.01, *p < 0.05, NS not significant.

To determine the potential clinical significance of RSL3 in MDS, we first treated peripheral blood mononuclear cells (PBMCs) from MDS patients with RSL3. Our results showed that RSL3 had an inhibitory effect in mononuclear cells from MDS patients but almost no effect in cells from the healthy counterparts (Fig. 6A–D). In clinical, resistance to HMA such as azacitidine (AZA) and decitabine (DAC) leads to treatment failure in MDS patients. Given the advantage of RSL3 in decreasing the MDS cells, we tested that whether RSL3 can synergize with HMA via combining RSL3 and DAC to treat MDS cells. The results showed that RSL3 had an obvious synergistic effect with DAC in MDS cell lines (Fig. 6E, F). Taken together, these results indicated that RSL3 inhibited the cell viability of MDS patients and was promising in combination with HMA agents to treat MDS cells.

## Discussion

In clinical, HMA such as azacytidine and decitabine have been proven to benefit the survival of MDS patients. However, many patients develop drug resistance [37]. Thus, the development of novel therapy including single reagents or drug combinations to treat MDS is in urgent need. Herein, we found that RSL3 inhibited proliferation of MDS cells via mediating MYB degradation and resulting in inducing cell apoptosis. More importantly, RSL3 had a synergistic effect with decitabine to treat MDS. Our findings demonstrated novel roles and related mechanisms of RSL3 in MDS and provided a promising choice for MDS patients.

RSL3 is widely used as a ferroptosis inducer. In colorectal cancer, RSL3 triggers ferroptosis by promoting ROS accumulation and increasing the cellular labile iron pool [38]. RSL3 was also found to significantly decrease prostate cancer cell viability, growth, migration and invasion. Importantly, it had no obvious side effects [39]. These studies showed the anticancer effect of RSL3 dependent on ferroptosis. However, we found that RSL3 decreased MDS-L and SKM-1 cells’ proliferation via inducing apoptosis instead of ferroptosis. The rescue assays during which only apoptosis inhibitors, not ferroptosis inhibitors nor other cell death inhibitors, can rescue the proliferation inhibition induced by RSL3, supported our conclusion. Furthermore, RNA-seq data showed that RSL3 treatment affects the expression levels of several molecules involving ferroptosis pathway, which further confirms RSL3’s roles independent ferroptosis in MDS cells.

Cellular ROS accumulation is closely related to apoptosis [40]. In our study, a series of biochemistry assays and ROS scavenger (NAC) rescue assay demonstrated that RSL3-induced cell apoptosis via increasing ROS accumulation. Clinically, repeated blood transfusion, inefficient erythropoiesis, and abnormal hepcidin expression cause iron overload in 50–80% of MDS patients [41]. Iron overload in these patients leads to an increase in ROS levels and induces apoptosis in bone marrow mesenchymal stromal cells, which can be partially reversed by treatment with iron chelators or antioxidants [42, 43]. A study showed that iron overload in patients with MDS caused ROS-dependent erythrocyte apoptosis [44]. DFO (an iron chelator) partially blocked RSL3-induced cell death. Therefore, RSL3-induced ROS-dependent apoptosis could be mediated by elevated Fe^2+^ levels. Through deep analysis of transcriptome sequencing data, we found that RSL3 increased ROS levels via decreasing MYB expression levels. The finding that MYB regulates ROS levels is consistent with previous study [16]. Interestingly, ROS levels also affected MYB expression (Supplemental Fig. 1B), indicating there was regulation loop existed.

Many studies have reported that abnormal MYB expression affects cancer cell expansion, differentiation, and apoptosis. MYB was associated with poor prognosis through inhibition of apoptosis in colorectal cancer [45]. Defects in the acetyltransferase p300 were found to promote MYB expression to accelerate MDS progression [46]. Most importantly, our previous work showed that MYB, as a transcription factor, promoted the malignant progression of MDS [4]. Herein, we found that RSL3 treatment decreased MYB protein levels in both dose-dependent and time-dependent manners. Importantly, RSL3 can directly bind to MYB protein and protect its thermal degradation. Even though MYB mRNA levels also decreased after RSL3 treatment, it was possible that RSL3 decreased MYB protein levels before its mRNA levels for that MYB can transcriptionally regulate itself [47]. Our findings demonstrated that RSL3 acted as MYB inhibitor in MDS cells, and it is worth to explore whether this mechanism is conserved in other cells, especially in MYB-induced cancer.

It was reported that venetoclax (VEN), a selective Bcl-2 inhibitor in combination with HMAs improved efficacy for multiply relapsed/refractory patients with MDS [48]. In our study, RSL3 decreased expression levels of MYB and Bcl-2, and RSL3 not only inhibited MDS cell proliferation as a single drug but also had synergistic effects with DAC to treat MDS cells, indicating promising clinical applications of RSL3 in the future. Unexpectedly, the results in MDS-L cells are more obvious than SKM-1 cells, which may be related to their different origins [49].

**Fig. 7:**
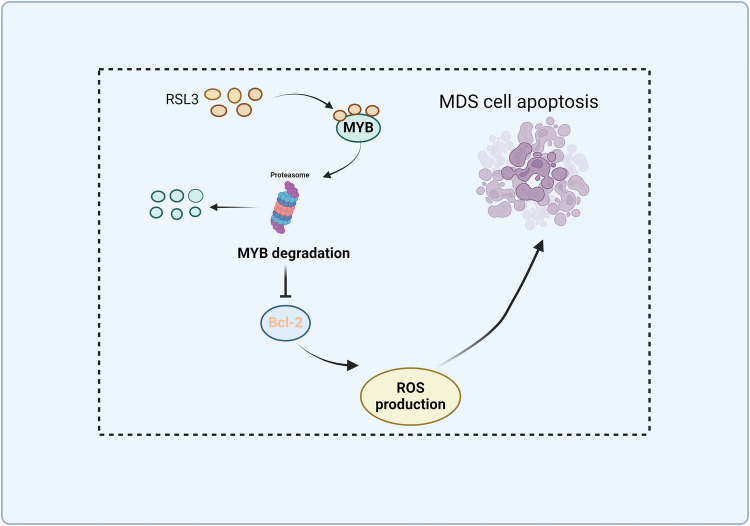
A schematic diagram of the proposed anti-MDS activity of RSL3. RSL3 directly targets MYB protein leads its degradation through proteasome, then leads to decreased Bcl-2 expression and increased ROS production further to promote MDS cell apoptosis.

In conclusion, our data revealed that RSL3 is a promising compound to inhibit MDS cell growth and overcome hypomethylating agent resistance. Mechanistically, RSL3 inhibits MYB to induce apoptosis by downregulating Bcl-2 expression. And we found that MYB could be a direct drug target of RSL3 and RSL3 is a potent MYB inhibitor that can be used to treat MDS and other hematopoietic diseases (Fig. 7).

### Supplementary information

Supplemental material

## Data Availability

The datasets generated and/or analysed during the current study are available from GSE256525.
