# Supplementary material for: RSL3 enhances ROS-mediated cell apoptosis of myelodysplastic syndrome cells through MYB/Bcl-2 signaling pathway
Source: Cell Death Dis. 2024 Jul 2;15(7):465. doi: 10.1038/s41419-024-06866-5 (PMC11219730; doi:10.1038/s41419-024-06866-5)
Supplement: Supplementary file 1 — Supplemental material [file 41419_2024_6866_MOESM1_ESM.pdf]

Figure S1

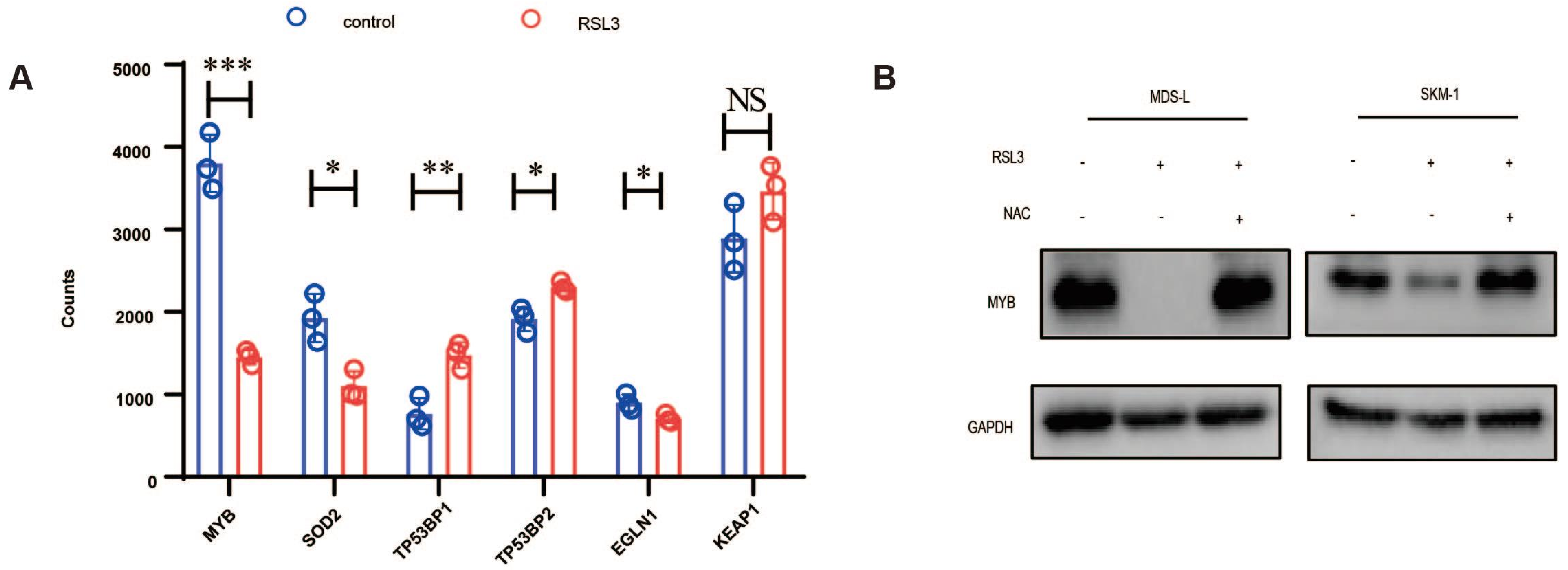

A MYB was downregulated and ferroptosis-associated genes like EGLN1 and KEAP1 had no significant changes by RNA seq.
B Western blotting of MYB in MDS cells treated with RSL3 alone or in combination with NAC.

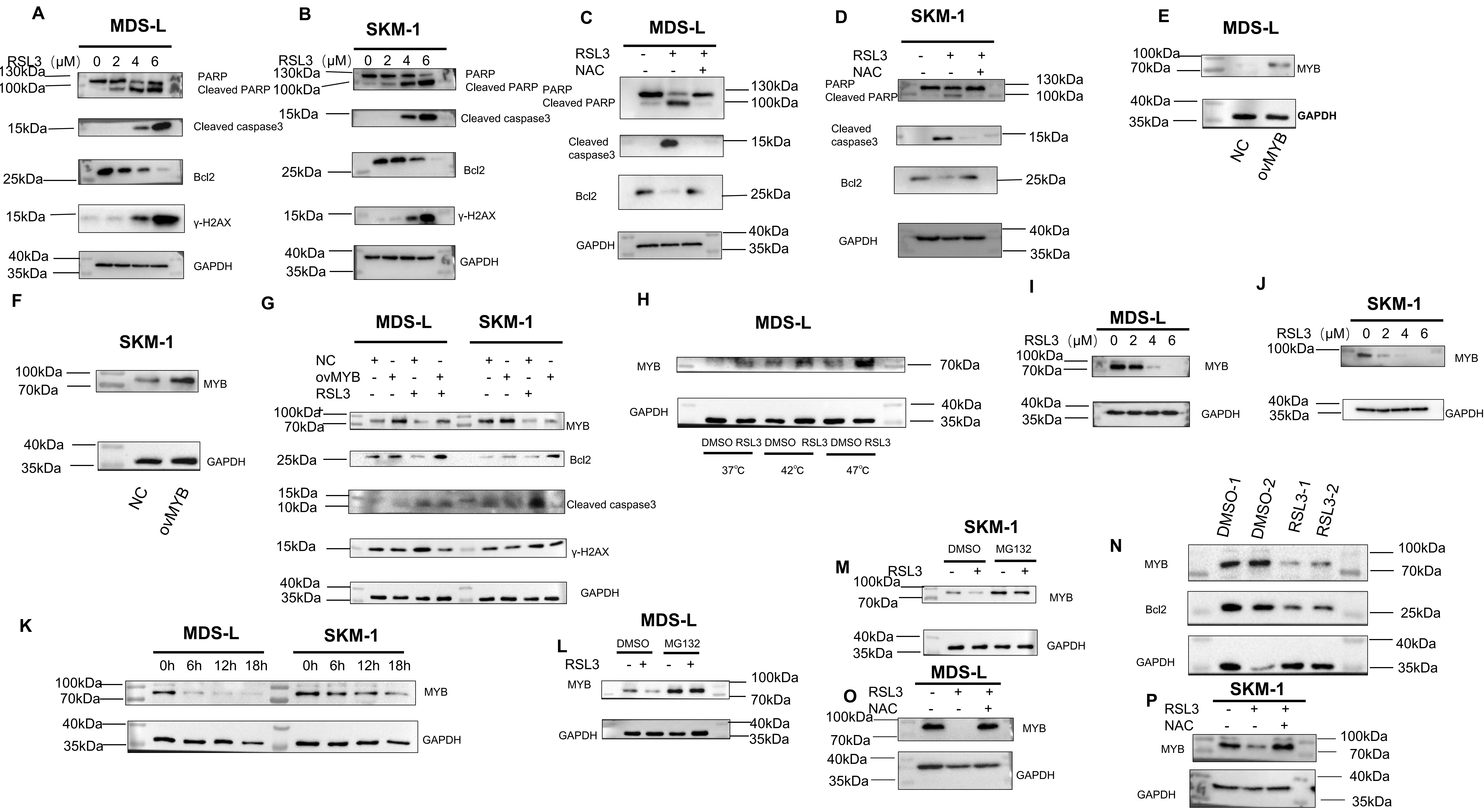
A
MDS-L
RSL3 (μM) 0 2 4 6
PARP
Cleaved PARP
Cleaved caspase3
Bcl2
γ-H2AX
GAPDH
B
SKM-1
C
MDS-L
RSL3
NAC
D
SKM-1
E
MDS-L
MYB
NC
ovMYB
F
SKM-1
G
ovMYB
H
DMSO RSL3 DMSO RSL3 DMSO RSL3
37℃
42℃
47℃
I
J
K
0h 6h 12h 18h
L
MG132
M
N
DMSO-1
DMSO-2
RSL3-1
RSL3-2
O
P
130kDa
100kDa
70kDa
40kDa
35kDa
25kDa
15kDa
10kDa
